# Neurons' Short-Term Plasticity Amplifies Signals

**DOI:** 10.1371/journal.pbio.0040240

**Published:** 2006-06-20

**Authors:** Mason Inman

Animals' neurons, and the synapses that connect them, are constantly changing. This plasticity is thought to underlie learning and memory. Take the rat in the maze. As he learns to navigate a new environment, familiarity with the space is reflected in the neuronal activity of a small almond-shaped brain structure called the hippocampus. Neurons in the hippocampus are generally quiescent. But when the rat meanders into a spot that a specific neuron prefers, called its “place field,” the neuron responds with high-frequency bursts of spikes. As the rat's familiarity with the maze increases over only a few minutes, so does the reliability by which hippocampal neurons respond to their preferred place. This short-term experience modifies the neurons' responses, and very likely the synapses, although the synaptic mechanisms of short-term plasticity in this context have not been fully described.

A new study takes a step forward in understanding the most basic level of this process: the short-term plasticity at hippocampal synapses that result from processing incoming signals resembling place-field responses. The researchers, Vitaly Klyachko and Charles Stevens, discovered a novel short-term plasticity mechanism by which excitatory and inhibitory synapses can selectively amplify high-frequency bursts.

For the study, the researchers used slices of the rat's hippocampus, focusing on cells from two particular regions, called CA1 and CA3, known for their role in encoding information about the animal's position. The researchers recorded long series of this firing activity, which they then used to stimulate two classes of hippocampal neurons: excitatory neurons, whose function is to spur neurons downstream to fire; and inhibitory neurons, which suppress neurons downstream.

In the hippocampus, these neurons form basic circuit elements, among which a “feed-forward loop” is one of the most common. In its simplest form, these loops feature an excitatory neuron connected to both an inhibitory neuron and an output neuron, and the inhibitory neuron is also connected to the output neuron. In this simple triangular network, incoming signals trigger both the excitatory and inhibitory neurons at once, and then the inhibitory neuron activates its synapses with a delay of a few milliseconds. From the output neuron's point of view, the incoming excitatory signals are closely followed by the inhibitory ones.

Several previous studies that tried to sort out how these neurons function during processing of incoming signals that resemble natural activity failed to produce coherent outputs from the neurons. These incoherent outputs may have resulted from the fact that the neurons were held at room temperature; as Klyachko and Stevens had shown before, short-term plasticity works differently at room temperature than at body temperature. To avoid the temperature problem in this study, Klyachko and Stevens held the brain slices at near body temperature.

With short-term plasticity, a synapse's response to any one signal depends on the signals it received in the previous few seconds. Synapses can sense when they're receiving a high number of impulses per second—that is, a high-frequency signal. Klyachko and Stevens found that, as long as the incoming signal was above a certain average rate, around 10 Hz, then the synapses would flip from a baseline state to an “active” state. The excitatory synapses became more excitatory, amplifying incoming signals. The inhibitory synapses responded oppositely, damping down their activity. Surprisingly, for any signals with higher frequency, these synapses' responses stayed constant even when the incoming signal rose to much higher frequencies, such as 100 Hz. The researchers also found that the excitatory and inhibitory synapses had mirror-image responses: when the excitatory synapses amplified a specific portion of a signal, the inhibitory synapses damped down their response at the same time.

When these two types of cells are wired together in a feed-forward loop, the researchers found that the excitatory and inhibitory synapses acted in concert, filtering out low-frequency signals while amplifying high-frequency signals. Thus, the study shows a function for the hippocampus's feed-forward loops not seen in earlier studies. It also shows a new role for inhibitory synapses: amplifying signals.

In this study, hippocampal neurons used short-term plasticity to filter neuronal signals for high-frequency events that encode important information for the animal. As the authors argue, this plasticity could also play a widespread role in information processing in the brain. Short-term plasticity may provide the mechanism by which animals' quickly changing brains help them navigate and comprehend the world.

## 

**Figure:**
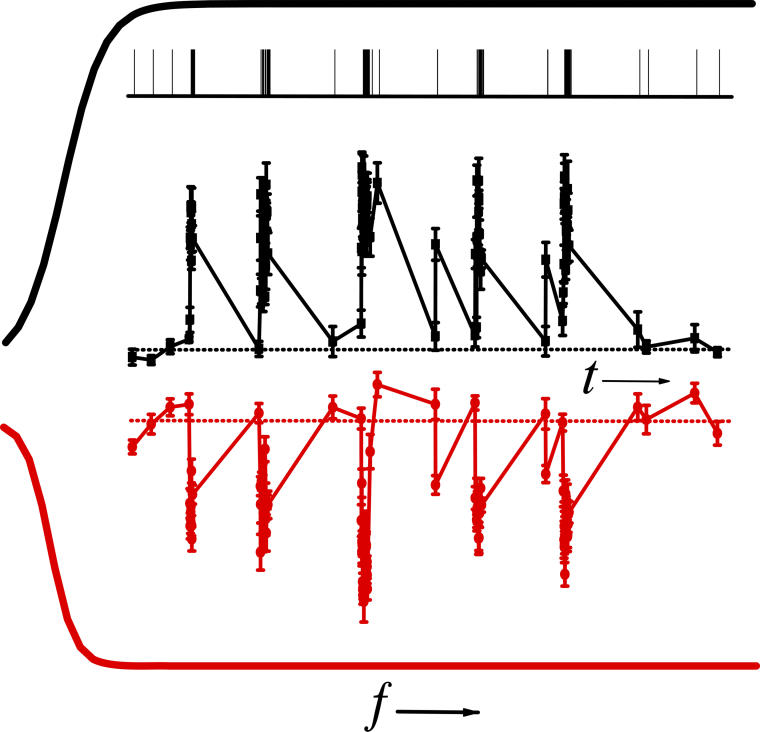
Hippocampal synapses work as an adaptive filter.

